# Unexpected Foreign Body Insertion Presenting as an Abdominal Mass in an Adolescent: A Case of Underlying Psychological Distress

**DOI:** 10.7759/cureus.85790

**Published:** 2025-06-11

**Authors:** El Atrache Hanae, Amara Ayoub, Zaari Najlae, Abdelouhab Ammor, Houssain Benhaddou

**Affiliations:** 1 Pediatric Surgery, Mohammed VI University Hospital Center, Oujda, MAR

**Keywords:** abdominal mass, anxiety and depressed mood, pediatric laparoscopic surgery, psychological distress, unexpected foreign body

## Abstract

We report the case of a 13-year-old female who presented with a painful, inflammatory mass in the left hypochondrium. Imaging studies revealed multiple subcutaneous foreign bodies, including metallic fragments and non-metallic objects. Surgical exploration under general anesthesia led to the extraction of approximately 20 metal wire fragments, glass debris, a pen tube, and a lollipop stick. Psychiatric evaluation disclosed a diagnosis of non-suicidal self-injury (NSSI) associated with emotional distress, social phobia, and a history of bullying. This case highlights the importance of a multidisciplinary approach combining surgical, psychiatric, and psychosocial support in adolescents with somatic presentations of psychological suffering. Early recognition of NSSI and prompt intervention are crucial to prevent complications and address underlying psychopathology.

## Introduction

Foreign body insertion is a rare but clinically significant manifestation of non-suicidal self-injury (NSSI), particularly in adolescents experiencing psychological distress.

NSSI is defined as the deliberate, self-inflicted damage of body tissue without suicidal intent and for purposes not socially sanctioned. It is most commonly seen in adolescents and may serve functions such as emotional regulation, coping with distress, or expressing internal pain. While cutting, scratching, or burning are the most frequent forms, other methods (including the insertion of foreign bodies) are less common and may be overlooked due to their atypical presentation and the patient's reluctance to disclose the behavior.

While cutting and burning are more common forms of NSSI, deliberate insertion of foreign bodies into subcutaneous tissues represents a less frequent but potentially dangerous behavior that may lead to severe infections, abscess formation, or systemic complications [1,2].

This report discusses a unique case of a young adolescent presenting with an inflammatory abdominal mass, ultimately found to be the result of self-inflicted insertion of multiple foreign bodies. The case underscores the importance of a high index of suspicion for NSSI in adolescents with unexplained wounds or masses, particularly when emotional or behavioral concerns are present. Additionally, it highlights the critical role of imaging, surgical intervention, and psychiatric evaluation in managing such cases.

## Case presentation

A 13-year-old female with no significant prior medical history was brought to the emergency department by her mother due to a seven-day history of progressively enlarging, painful, erythematous swelling in the left hypochondrium. The mother reported that the lesion had initially appeared as a small puncture wound but had rapidly evolved into a tender, fluctuant mass with serous discharge. There was no history of trauma, recent travel, or animal bites.

The patient comes from a family with no reported history of psychiatric disorders. However, the family dynamic was marked by communication difficulties and emotional distance.

**Figure 1:**
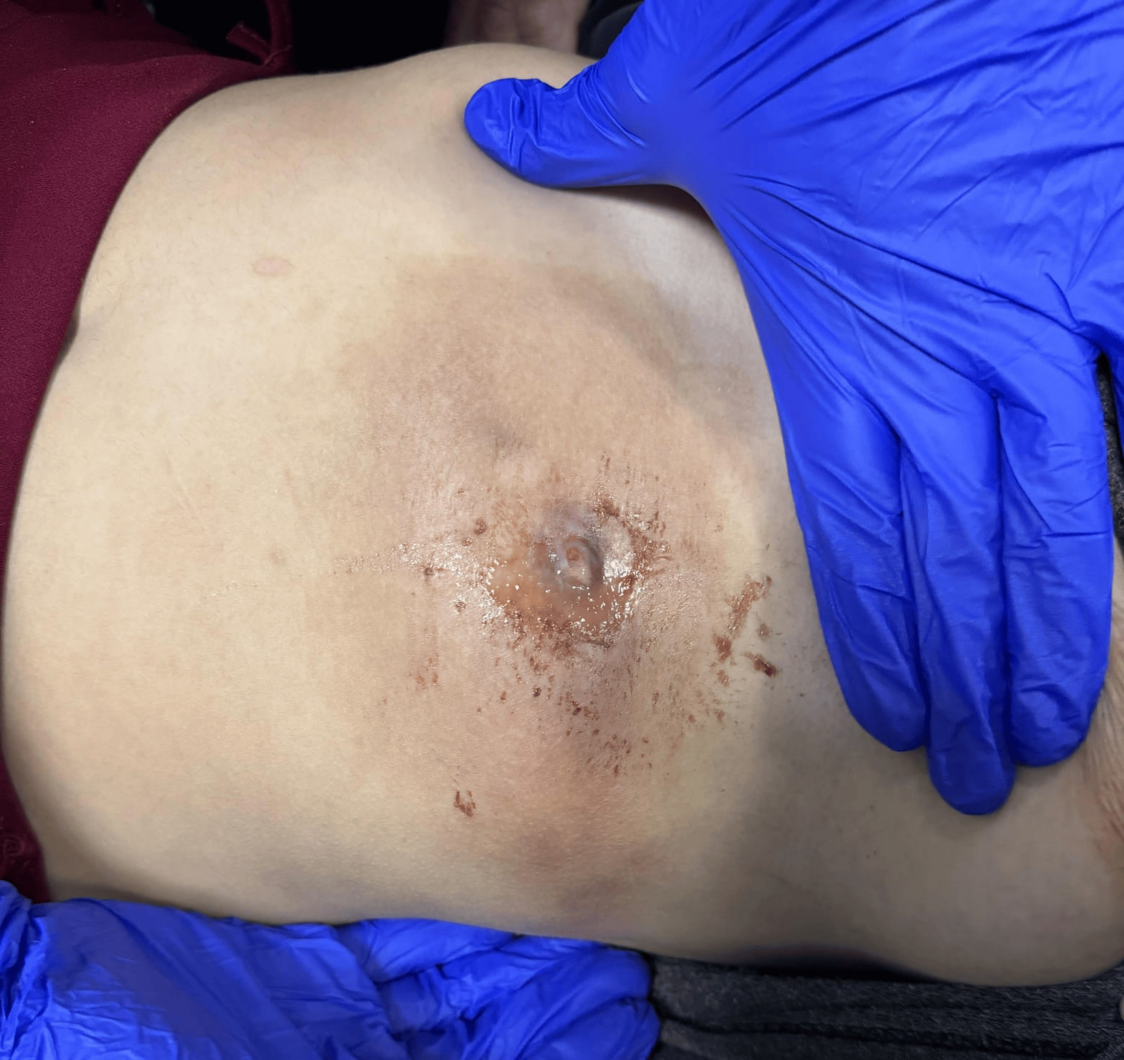
Clinical examination revealed a palpable mass in the left hypochondrium, with a central punctum exhibiting serous discharge.

On physical examination, the patient was afebrile and hemodynamically stable. A 5 × 4 cm warm, erythematous, indurated mass was observed, with a central punctum oozing serous fluid (Figure 1). There was no lymphadenopathy or signs of systemic infection. Upon further inspection, multiple small linear scars were noted on the patient’s forearms and thighs.

**Figure 2:**
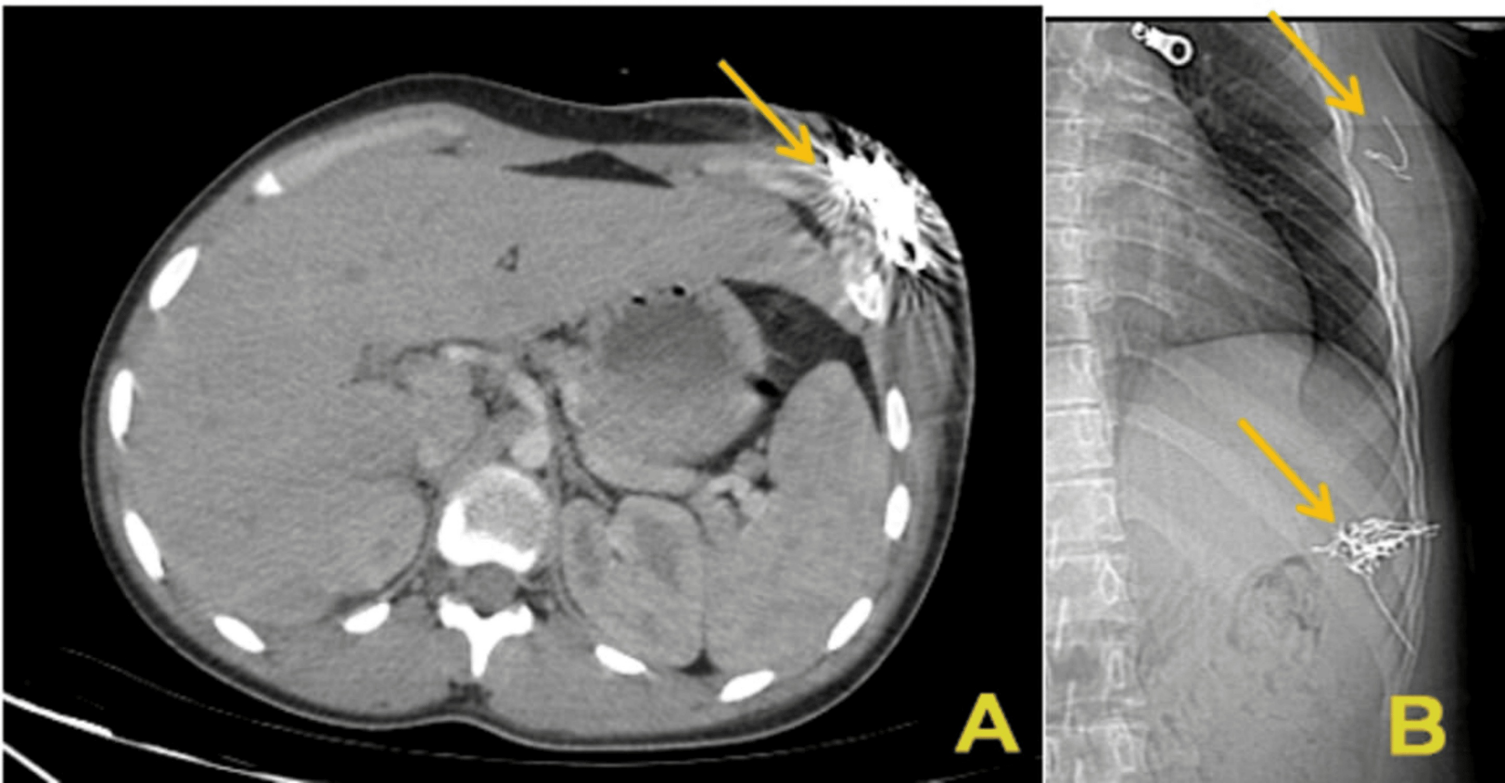
CT image (A) and radiographic image (B) showing the location of the foreign bodies, indicated by yellow arrows. A) Axial contrast-enhanced CT scan of the abdomen showing a cluster of hyperdense linear structures in the subcutaneous tissue of the left hypochondrium (yellow arrow), consistent with metallic foreign bodies. Beam-hardening artifacts are visible around the area, indicating the metallic nature of the objects. B) Lateral radiographic view of the thoracoabdominal region highlighting two separate collections of foreign bodies: one located in the left hypochondrium and another in the subcutaneous tissue of the left breast (yellow arrows). The radio-opaque nature and clustering suggest multiple metallic fragments, most likely inserted subcutaneously.

Imaging studies revealed multiple hyperechoic foreign bodies on ultrasound, with posterior acoustic shadowing surrounded by hypoechoic inflammatory tissue. Computed tomography (CT) confirmed the presence of numerous metallic fragments, likely wire fragments, which caused significant beam-hardening artifacts. These were distributed over a 76 mm span within the subcutaneous tissue. Additionally, a second cluster of foreign bodies was identified in the left breast, raising concerns about other self-insertion sites (Figure 2).

**Figure 3:**
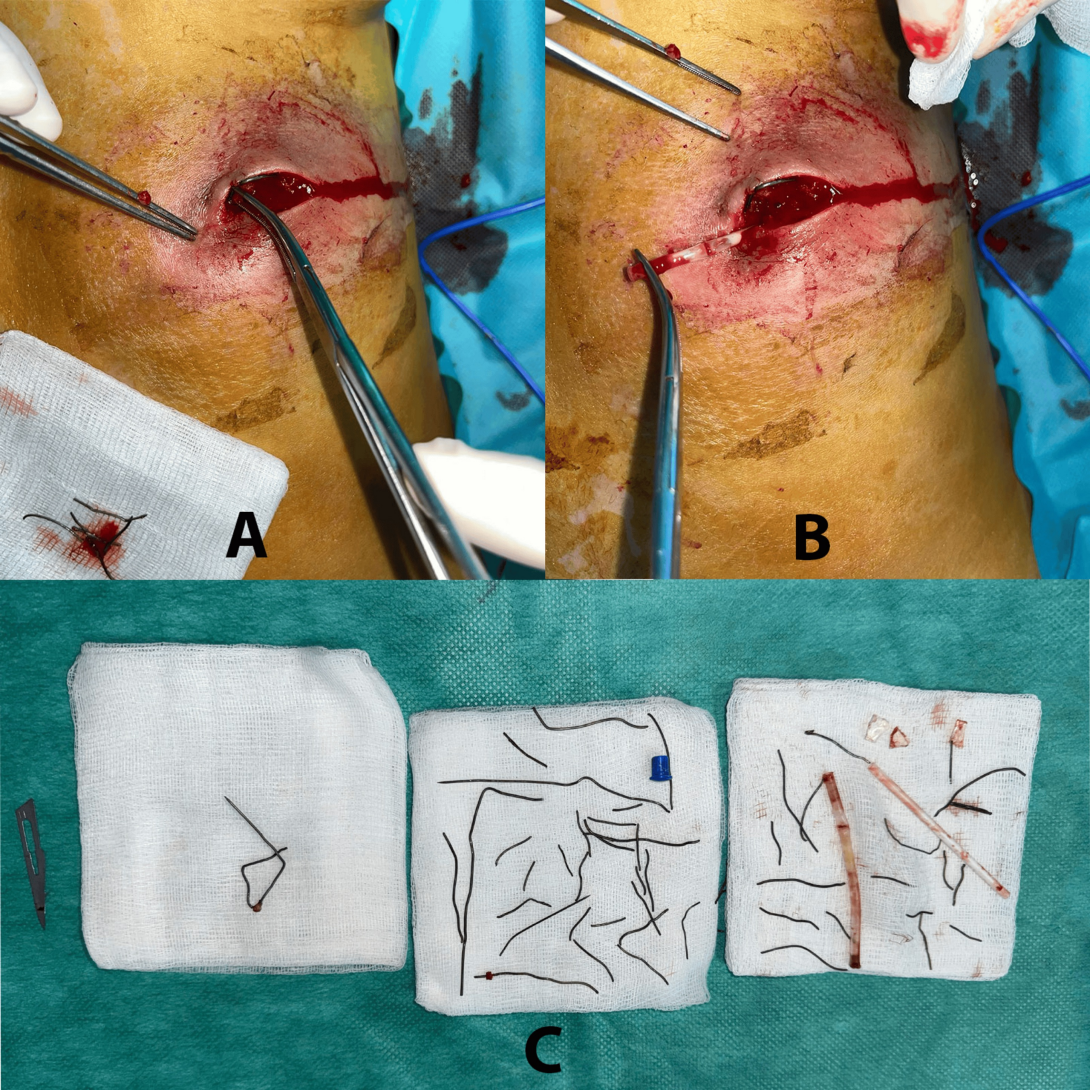
Intraoperative image of foreign body extraction (A and B). Fragments of metal wire, glass shards, a plastic tube, and other heterogeneous objects placed on sterile gauze pads after complete removal (C).

Surgical exploration was conducted under general anesthesia. A 4 cm incision was made over the affected area, revealing purulent material and multiple embedded foreign bodies. Approximately 20 metallic wire fragments ranging in length from 5 mm to 25 mm were extracted, along with glass shards, a plastic pen tube, and a lollipop stick (Figure 3). Intraoperative fluoroscopy confirmed the complete removal of all radiopaque materials. The wound was irrigated with saline and betadine and then closed using interrupted sutures.

In the postoperative period, the patient admitted to a six-month history of deliberate self-insertion of foreign objects as a coping mechanism for emotional distress. She denied any suicidal intent but described persistent feelings of worthlessness, social isolation as a result of bullying at school, and anxiety in social contexts consistent with social phobia. She also reported a history of superficial cutting on her arms and thighs.

A formal psychiatric evaluation confirmed a diagnosis of NSSI in the context of adjustment disorder with mixed anxiety and depressed mood. The patient was referred for cognitive behavioral therapy (CBT) and was enrolled in a structured dialectical behavior therapy (DBT) program, with a focus on emotional regulation and distress tolerance.

The wound healed without infection over a period of three weeks. At the six-month follow-up, the patient reported a significant reduction in self-harming behaviors and an overall improvement in mood through therapy. Family counseling was also initiated to address underlying interpersonal stressors.

Informed written consent was obtained from the patient’s legal guardian both for the surgical intervention and for the inclusion of the clinical details and images in this published case report.

## Discussion

This case illustrates an uncommon but clinically significant manifestation of NSSI, in which the subcutaneous insertion of multiple foreign bodies mimicked infectious or neoplastic pathology. Although NSSI affects approximately 10-20% of adolescents, the implantation of foreign objects under the skin remains rare [3].

From a psychopathological perspective, NSSI serves several functions, including emotional regulation, self-punishment, and interpersonal signaling [4]. In this patient, emotional dysregulation and social rejection appeared to be the primary triggers, consistent with studies that link NSSI to experiences of bullying and peer victimization [1]. The abdominal location of the self-harm, as opposed to more visible sites like the limbs, may reflect shame or fear of discovery, which in turn contributes to delayed diagnosis and management [5].

Diagnostically, such cases are challenging due to vague clinical histories, patient concealment, and non-specific symptoms such as pain and swelling that can mimic abscesses or soft tissue tumors. In addition, certain materials, such as plastic or organic substances, may be radiolucent, making them undetectable on standard radiographs. CT remains the gold standard for identifying metallic or high-density foreign bodies, while ultrasound may assist in detecting radiolucent objects [6].

In clinical practice, the presence of unexplained localized soft tissue masses in adolescents, particularly when accompanied by vague or inconsistent histories, should raise suspicion for underlying NSSI. This is especially relevant when physical signs such as linear scars, behavioral indicators of emotional distress, or a history of social withdrawal are present. In such cases, early psychiatric evaluation and targeted imaging are crucial to uncover concealed self-injurious behaviors and prevent further harm. This case illustrates the importance of maintaining a high index of suspicion and reinforces the need for multidisciplinary collaboration in similar presentations.

In this case, intraoperative fluoroscopy was essential to ensure the complete removal of all radiopaque materials. Prompt surgical intervention is recommended to avoid complications such as infection, migration, or vascular injury. The use of intraoperative imaging, such as fluoroscopy or ultrasound, can minimize the risk of retained fragments [7].

Psychiatric care is a key component of comprehensive management. CBT and DBT are considered first-line treatments for NSSI, focusing on emotional regulation and coping strategies [8]. Family involvement is also critical to address underlying relational stressors.

Given that recurrence rates of NSSI can reach up to 50% within one year, long-term psychiatric follow-up is strongly recommended [9]. Furthermore, school-based interventions may help reduce social isolation and bullying, which are frequently reported among adolescents who engage in NSSI.

## Conclusions

This case underscores the importance of considering NSSI as a differential diagnosis in adolescents presenting with unexplained soft tissue masses or inflammatory lesions, particularly when accompanied by vague histories and behavioral signs of emotional distress. The unusual presentation in the left hypochondrium, mimicking an abscess or soft tissue tumor, delayed the diagnosis and required a thorough surgical exploration. The extraction of multiple metallic wires, glass fragments, and non-metallic foreign bodies highlights the complexity and potential risks of this form of self-injury. Successful management in this case required a coordinated multidisciplinary approach, including prompt surgical intervention, imaging guidance, psychiatric evaluation, and ongoing psychosocial support. Preoperative assessment, intraoperative fluoroscopic control to ensure complete removal of foreign materials, and careful postoperative care contributed to an uncomplicated physical recovery.

Psychiatric follow-up revealed the underlying emotional distress, social anxiety, and history of bullying that triggered the NSSI behavior. The integration of CBT, DBT, and family counseling played a critical role in addressing the root causes and reducing the recurrence of self-harm. In conclusion, early recognition of NSSI and timely integrated intervention are essential not only for preventing physical complications such as infection or retained foreign bodies but also for treating the underlying psychopathology. This case illustrates how clinical vigilance, surgical precision, and comprehensive mental health support can collectively improve the long-term outcome for adolescents engaged in self-injurious behavior.
